# The co-evolution of networks and prisoner’s dilemma game by considering sensitivity and visibility

**DOI:** 10.1038/srep45237

**Published:** 2017-03-24

**Authors:** Dandan Li, Jing Ma, Dun Han, Mei Sun, Lixin Tian, H. Eugene Stanley

**Affiliations:** 1 College of Economics and Management, Nanjing University of Aeronautics and Astronautics, Nanjing, Jiangsu 211106, China; 2 Center for Polymer Studies and Department of Physics, Boston University, Boston, MA 02215, USA; 3 Nonlinear Scientific Research Center, Jiangsu University, Zhenjiang, Jiangsu, 212013, China; 4 School of Mathematical Science, Nanjing Normal University, Nanjing, Jiangsu, 210042, China

## Abstract

Strategies adopted by individuals in a social network significantly impact the network, and they strongly affect relationships between individuals in the network. Links between individuals also heavily influence their levels of cooperation. Taking into account the evolution of each individual’s connection, we explore how sensitivity and visibility affect the prisoner’s dilemma game. The so-called ‘sensitivity’ and ‘visibility’ respectively present one’s self-protection consciousness and the ability of gaining information. We find that at moderate levels of player sensitivity cooperative behavior increases, but that at high levels it is inhibited. We also find that the heterogeneity of the weight of individuals at the end of the game is higher when sensitivity and visibility are increased, but that the successful-defection-payoff has less impact on the weight of individuals and on the relationship between the heterogeneity of the weight of individuals and the density of cooperators. This framework can be used to clarify the interaction mechanism between the micro-level of individual behavior and the macro-level of individual co-evolutionary processes.

Robert May has said that the most important unanswered question in evolutionary biology and in the social sciences in general is how cooperative behavior evolved and how it is maintained in human societies and in animal groupings^1^. The cooperative behavior of individuals is significantly affected by their mobility and interaction and especially by the complexity of their environment^2^,^3^,^4^. The task is to discover why some social interaction settings flourish with high levels of foresight and cooperation, while others are hindered by shortsighted insensitive behavior. In answering this question, game theory provides a powerful theoretical framework for studying individual-level motivation^5^,^6^,^7^. Game theory enables us to understand how cooperation can emerge and persist among even selfish individuals, and over the last decade evolutionary game theory using graphs and networks has attracted much attention^8^,^9^,^10^,^11^,^12^,^13^. The prisoner’s dilemma game (PDG) in particular has become a widely used model for investigating cooperation and cheating, with cooperation often emerging as a robust outcome in evolving populations^14^,^15^,^16^,^17^,^18^. The standard prisoner’s dilemma game describes the competition between cooperation and defection^19^,^20^,^21^,^22^,^23^. Of greater interest is what happens when individual gain and loss depend heavily on the actions of others^24^.

Although cooperation is ubiquitous in social life and an important topic in a wide variety of economic interactions, field data rarely enable us to discriminate among competing theories. Although our understanding of cooperative behavior among humans is thus limited, carefully designed laboratory experiments may enrich our knowledge of human cooperation^25^. In many real-world social systems, people have access to only their own information and are unable to access information about their neighbors^26^. Researchers at Yale University performed rigorous laboratory experiments that explored how initial wealth inequality and the structure of the social network influence the evolution of inequality. Their results indicate that increasing an individual’s visibility discourages cooperation^27^,^28^, that inequality affects rich and poor individuals differently, and that an individual’s visibility dynamically contributes to inequality. The complexity contributed by human mobility and interaction affects the evolution of closeness between individuals over time. In general, people increase their connections with partners who bring benefits and reduce their connections with those who do not. Because the constantly changing state of social networks leads to changes in personal influence, the network of connections between individuals is time-varying. Characterizing and modeling time-varying networks is still an open and active area of research. The heterogeneity of social ties is a key ingredient in social networks and plays a crucial role in individual cooperation. We still do not have an understanding of the mechanism driving the formation of heterogeneous social ties and their effect on dynamical phenomena that takes into account the time-varying nature of networks.

In an adaptive dynamical environment, choosing an advantageous strategy is the key factor in winning. A chosen strategy can significantly affect relationships with other individuals, but the closeness among individuals also heavily influences their chosen level of cooperation. To understand the interaction mechanism between the micro-level of individual behavior and macro-level of individual co-evolutionary processes, we construct a model that takes into consideration the evolution of the links of individuals and explore how sensitivity and visibility affect the prisoner’s dilemma game. Although we find the sensitivity and visibility of individuals can persuade more players to adopt a cooperative strategy and increase individual weight heterogeneity, we also find that a higher level of sensitivity has a negative impact on cooperative behavior. We also find that the successful-defection-payoff has less impact on individual weight heterogeneity and on the relationship between individual weight heterogeneity and cooperator density.

## Results

**Figure 1:**
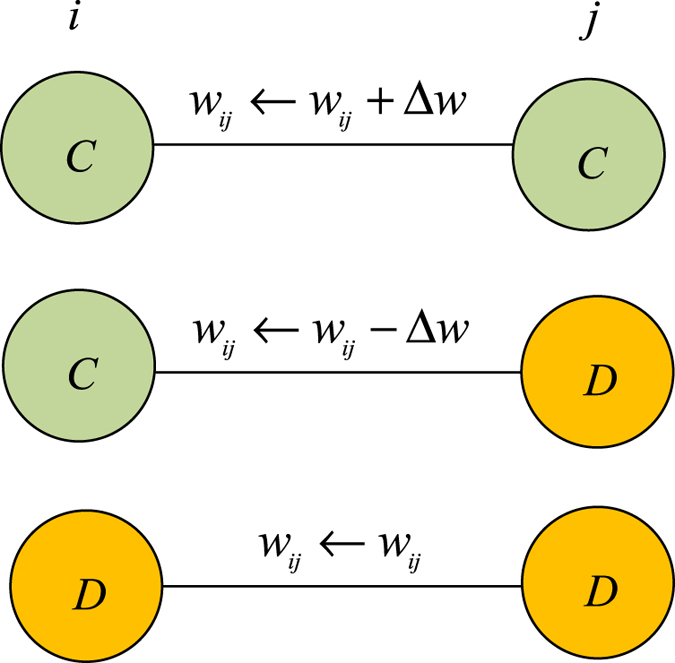
The edge weight evolution in the prisoner’s dilemma game. C and D represent cooperator and defector, respectively. The edge types between any two players are divided into three relations: cooperation-cooperation, cooperation-defection, and defection-defection, which are marked as C ↔ C, C ↔ D and D ↔ D, respectively. The edge weight w_ij_ between any two player i and j changes with their respective strategies.

We distinguish the edge types between any two players as cooperation-cooperation, cooperation-defection, or defection-defection, and we mark them *C* ↔ *C, C* ↔ *D*, and *D* ↔ *D*, respectively. Here we adopt edge weight *w*_*ij*_ to measure the closeness of two players. A large edge weight in general indicates a better relationship between the two players, and a small one the opposite. The edge weight *w*_*ij*_ evolves with player strategy. Note that a win-win strategy promotes the relationship between two individuals. The edge weight increases when both ends of an edge choose a cooperation strategy, but when a cooperator connects with a defector, the cooperator loses heavily and the closeness is significantly weakened. The evolution of closeness is


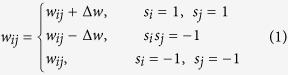


where Δ*w* is the variable used to reflect the individual’s sensitivity awareness. When the value of Δ*w* is large, the players are extremely sensitive to changes in their payoff. The result is that when a cooperator connects with a defector, the closeness decreases heavily in the next game round. In particular, the link between players *i* and *j* breaks when *w*_*ij*_ ≤ 0, and the player who receives the smaller payoff will choose to connect to another player. Note that self-connections and multi-edges are avoided. Figure 1 shows the evolution of edge weight between players *i* and *j*.

We consider the evolution of social dilemmas on a network of size *Z* = 1000. Initially the topology is a Erdos-Renyi random network with an average degree <*k*> = 6. Because the relationship between any two players differs, we set a weight 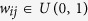 for every edge in the network (note that *U*(0, 1) stands for a uniform distribution). We then use the Monte Carlo method and perform a prisoner’s dilemma game^29^,^30^ in which each player *i* is initially designated with equal probability either a cooperator (*C*) or defector (*D*) (see Methods). To alleviate randomness, we obtain the equilibrium by averaging over 100 independent runs.

**Figure 2:**
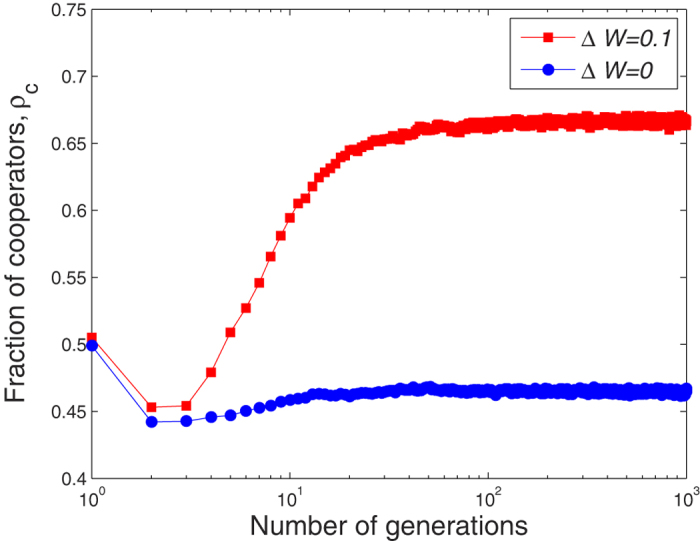
The fraction of cooperators *ρ*_*c*_ evolves with game process. We can find by comparison, the individuals’ rational behaviors make the opportunism behavior having a higher risk to be abandoned. Consequently, the density of cooperators *ρ*_*c*_ will greatly increase. Because of the interaction between payoff and closeness, the cooperators’ density and free-rider density would stabilize eventually. The evolutionary social dilemmas is on a network of size Z = 1000. Initially, the network topology is the Erdos-Renyi random network with the average degree 〈*k*〉 = 6. Other parameters are set to be *p* = 0.5 and *T* = 1.1.

Under a different sensitivity awareness Δ*w*, Fig. 2 shows how the fraction of cooperators *ρ*_*c*_ evolves as the game progresses. The unique Nash equilibrium in the prisoner’s dilemma game is defection-defection. The optimal Pareto configuration is cooperation-cooperation. Because cooperation brings an overall increase in benefit, the equilibrium of the optimal behavior of a single individual does not coincide with the Pareto optimum. We can find this through comparison. Although players often defect in hopes of obtaining a larger income, this weakens relationships and raises the probability that opportunistic defectors (free-riders) will be quickly abandoned, causing a significant decrease in their income. Figure 2 also shows that when this occurs the free-riders return to being cooperators and thus the density of cooperators increases. Because of the interaction between payoff and closeness, the densities of cooperators and free-riders eventually stabilize.

**Figure 3:**
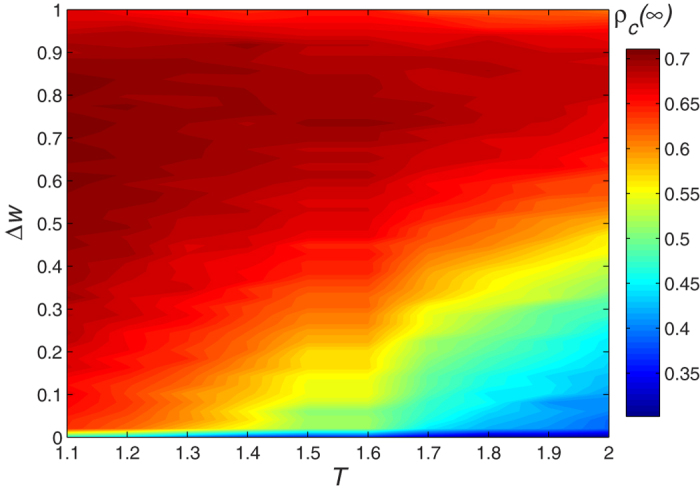
The final cooperators density *ρ*_*c*_(∞) changes with the Successful-Defection-Payoff *T* and individual sensitivity Δ*w*. Players are willing to take defection strategy when *T* is larger. However, with the increase of the sensitivity of the individual, the cooperators density in the whole social network becomes larger. Defectors with more neighbors may get a larger income, but in general defectors have fewer neighbors and most of them will also be defectors. This eventually reduces the total income of the defectors. Parameter is *p* = 0.5.

Although the successful-defection-payoff strategy *T* produces a higher return, the higher the sensitivity awareness Δ*w* of the players, the more quickly they change their relationships with each other. Thus when a player adopts a betrayal strategy, his links with cooperative players disconnect. Defectors with more neighbors may get a larger income, but in general defectors have fewer neighbors, most of which are also defectors. This eventually reduces the total income of the defectors. Figure 3 thus shows that as player sensitivity increases the density of cooperators in the social network also increases.

**Figure 4:**
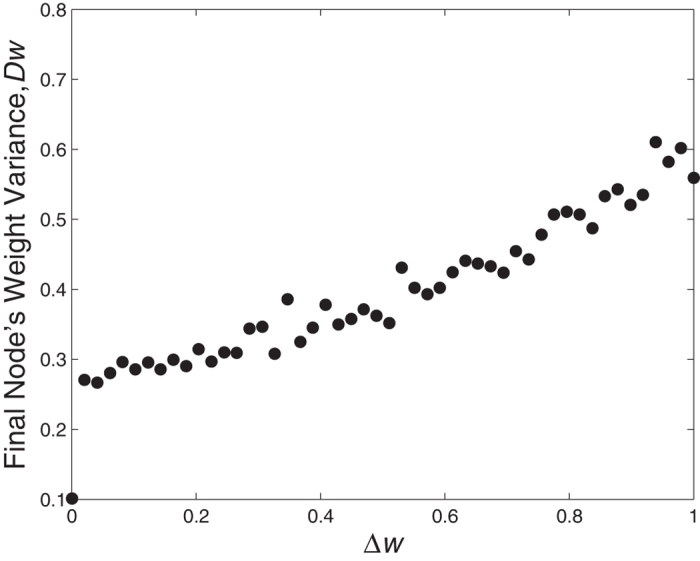
The network heterogeneity *D_w_* changes with the individuals’ sensitivity Δ*w*. We may easily observe that a larger value of Δ*w* leads to a larger *D_w_*. When the sensitivity of a player increases to a certain level, the edges connecting them to betrayers break easily. Parameters are *p* = 0.5 and *T* = 1.1.

The importance of the node within a system is its weight, and node weight is strongly associated with the weight of its edge. We calculate the weight of node *i* by adding up all the edge weights that link to its partners. Thus the weight of node *i* is 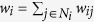, where *N*_*i*_ denotes the neighbor node sets. Variance informally measures how far a set of numbers is spread out from their mean, we here use 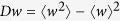 to measure the heterogeneity degree of the network, where 〈*w*^2^〉 is the mean of the square of the weight, 〈*w*^2^〉 is the square of the mean of the weight. Figure 4 shows that network heterogeneity increases as individual sensitivity Δ*w* increases. When the sensitivity of a player increases to a certain level, the edges connecting them to betrayers break easily. Although the betrayal strategy can bring greater benefits to an individual, changes in the network topology and node connectivity accelerate as player sensitivity increases. Eventually most of the cooperative players are linked with other cooperative players, and the closeness between the win-win players is enhanced. At the same time, players who choose defection have a low probability of connecting with other players. Thus network heterogeneity increases.

**Figure 5:**
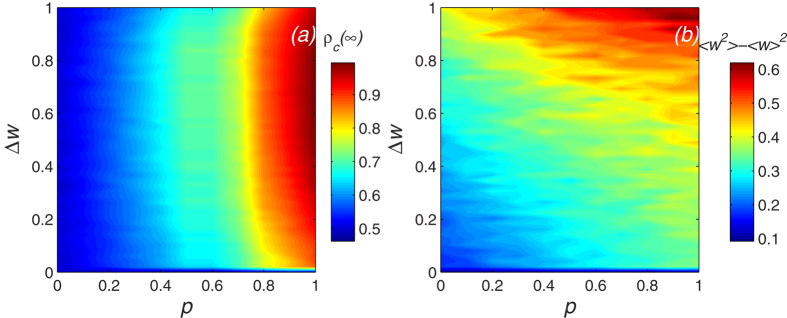
The final density of cooperators *ρ*_*c*_(∞) and level of network heterogeneity *D*_*w*_ = ⟨*w*^2^〉 − ⟨*w*^2^〉 change with the visibility levelp and the individuals’ sensitivity Δ*w*. We can get the value of *ρ*_*c*_(∞), and *D_w_* increases with the visibility level *p*. Thus the visibility facilitates the persistence of cooperation. Parameter is *T* = 1.1. (**a**) The change in final cooperator density *ρ*_c_(∞); (**b**) The change in network heterogeneity *D*_*w*_.

The visibility level of individuals in the network reflects the information flow. In a social network, the level of visibility is also an expression of the fairness of information acquisition. The greater the proportion of visible individuals, the greater will be the ability of the individuals to obtain information. When there is a high initial visibility level *p*, the final cooperator density *ρ*_c_(∞) is significantly higher than when there is a low initial visibility level. Figure 5(a) shows that visibility facilitates the persistence of cooperation. Note that when social relations are allowed to change and players can access more information, they are more likely to choose a cooperative strategy. Figure 5(b) shows that as the player visibility *p* increases, network heterogeneity slowly increases. At a higher visibility level *p*, players can obtain greater amounts of information from their neighbors. When a player can see that their income is lower than that of their neighbors, they change their current connection mode and cause an imbalance in weight distribution.

**Figure 6:**
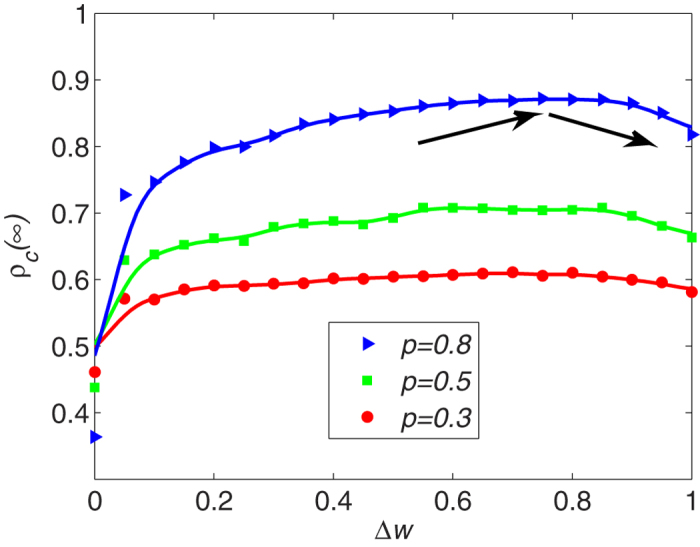
Under different visibility levels p, the density of cooperators changes with the individual sensitivity Δ*w*. The Δ*w* value significant affects the final heterogeneity level of the system. The density of cooperators *ρ*_*c*_(∞) increases first and then decreases with individual income sensitivity Δ*w*. Parameter is *T* = 1.2. Triangular, square and circular data points are the results of simulations. The line is obtained by using nonparametric regression method (The nonparametric regression method is described in methods parts).

Figure 6 shows different visibility levels *p* exhibiting qualitatively different behavior. The density of cooperators *ρ*_c_(∞) increases with individual income sensitivity Δ*w* irrespective of the *p* value. Note that when the sensitivity increases to a certain value, the final cooperator density decreases. The Δ*w* value significantly affects the final heterogeneity level of the system. When individual sensitivity Δ*w* increases to a certain level, small changes in individual payoff strongly impact the relationships among the players. Although it encourages players to connect to optimal “partners,” relationships between the individuals change rapidly. The network becomes “unstable.” It is unstable in terms of network topology and network connectedness. When network topology changes rapidly, players connect to new neighbors more quickly irrespective of the strategy the neighbor has adopted. However, here individuals may link to defectors more frequently, which causes more players to take the betrayal strategy and eventually decreases the proportion of cooperative players.

**Figure 7:**
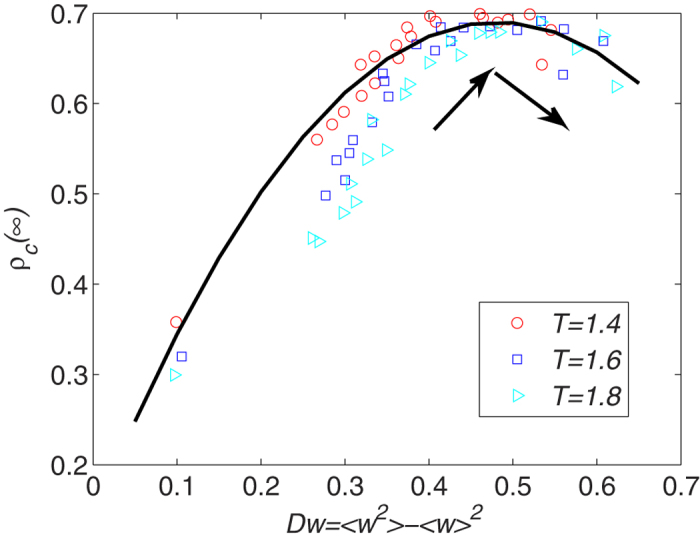
The relation between the final density of cooperators *ρ*_c_(∞) and the network heterogeneity *D_w_*. Δ*w* is taken from 0 to 1. We find that the density of cooperators first increases and then decreases with network heterogeneity under differing successful-defection-payoff *T* values. Other parameter is *p* = 0.5. Triangular, square, and circular data points are the results of simulations. The line is obtained by using nonparametric regression method (The nonparametric regression method is described in methods parts).

The final network heterogeneity *D_w_* reflects the differentiation of the system. A large *D_w_* value means that the distribution of node influence is more heterogeneous. In order to study the relation between *ρ*_*c*_(∞) and *D*_w_, we use the value of Δ*w* from 0 to 1 (the parameter interval is 0.01) in our model. We find that the density of cooperators first increases and then decreases with network heterogeneity under differing successful-defection-payoff *T* values. Figure 7 shows that some *D_w_* values facilitate cooperative behavior between selfish individuals. At the same time the sensitivity of players to their payoffs hinders cooperative behavior. The successful-defection-payoff *T* has less influence on the relation between the final network heterogeneity *D_w_* and the density of final cooperators *ρ*_*c*_(∞).

**Figure 8:**
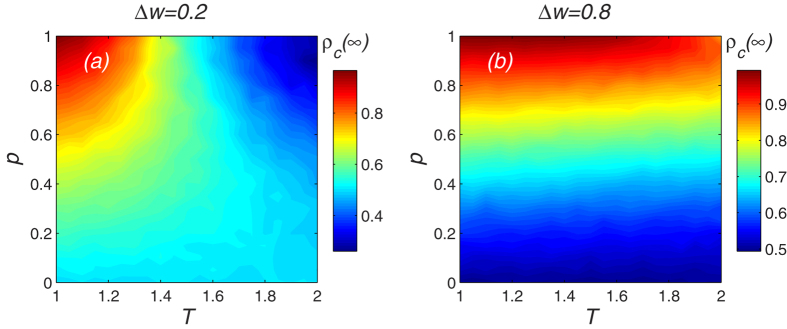
The density of cooperator evolves with the visibility level p and Successful-Defection-Payoff *T*. The cooperator density always increases as visibility level increases for a relatively small *T*, irrespective of individual sensitivity values. However, under a low individual sensitivity Δ*w*, the cooperator density decreases as visibility level increases for a large *T*. Parameter is *T* = 1.1. (**a**) Using a low individual sensitivity Δ*w* = 0.2; (**b**) Using a high individual sensitivity Δ*w* = 0.8.

We see that an individual cooperative behavior can be controlled by a player’s past experiences and present environment. Figure 8 shows how cooperative behavior evolves with the visibility level *p* and successful-defection-payoff *T*. Figures 8(a) and (b) show that cooperator density always increases as visibility level increases for a relatively small *T*, irrespective of individual sensitivity values. However when individual sensitivity is low Δ*w*, the cooperator density decreases as the visibility level increases for a large *T*. At the same visibility level and successful-defection-payoff *T* value, a larger Δ*w* promotes cooperation more effectively. Note that when Δ*w* is large enough, the value of *T* has little effect on the density of cooperators *ρ*_*c*_(∞), but when Δ*w* is small the value of *T* strongly influences the density of cooperators *ρ*_*c*_(∞).

## Discussion

To investigate some of the consequences of cooperation in the prisoner’s dilemma game, we have examined the effects of sensitivity and visibility. We find that although high player sensitivity promotes cooperative behavior, extremely high levels inhibit it. We also find that the heterogeneity of player weights in the final network is higher at higher levels of sensitivity and visibility, but that the successful-defection-payoff *T* has a smaller impact on the heterogeneity of player weights and on the relationship between heterogeneity of player weights and cooperator density. We have run our model using different parameter values, and we have found that the final results indicate a high level of robustness. The framework developed here could be used to clarify the interaction mechanism between the micro-level of individual behavior and macro-level of individual co-evolutionary processes.

To summarize, in a complex social network, choosing a strategy is important. A player’s chosen strategy strongly affects their relationship with other players, and the links between individuals strongly affect their decision of whether to cooperate. This study offers a new perspective on the prisoner’s dilemma game in a dynamic social network: how player sensitivity and visibility affect its evolution. Our analysis provides a framework for studying the co-evolution of games and strategies, and suggests that maintaining cooperation may be more difficult than previously thought. Although in some real-world systems restrictive assumptions may not be germane, our work sheds some light on the framework of evolutionary games in networks. Although here we focus on how links directly affect individual behavior, we do not consider how links *indirectly* affect individual behavior, i.e., we focus on how people influence others when there is a connection, irrespective of linking weight. Our model is nevertheless realistic. In general, conflicts of interest exist when there are connection, and vice versa. We here use a simple but practical model to show the evolution of human behavior. Further experimental research is needed to clarify our results, and we hope that our work will motivate work in that direction.

## Methods

### Prisoner’s dilemma game

We consider the prisoner’s dilemma game in a network *A* composed of *Z* players in which *A* = (*a*_*ij*_)_*z×z*_ is the adjacency matrix. When two players *i* and *j* interact, *a*_*ij*_ = 1, otherwise *a*_*ij*_ = 0. In the classical prisoner’s dilemma game, each player decides to be either a cooperator or a defector. When a cooperator links with a defector, the cooperator receives payoff *S* and the defector receives payoff *T* (we designate this scenario successful-defection-payoff). When both are cooperators, both receive payoff *R*, and when both are defectors, both receive payoff *P*. The prisoner’s dilemma game thus satisfies the conditions*: T* > *R* > *P* > *S* and *2R* > *T* + *S*^31^,^32^. When *R* > *P* mutual cooperation produces a higher payoff than mutual defection. When *T* > *R* and *P* > *S* the player with a defection strategy will obtain a higher payoff, regardless of what choice the opponent makes in a single round of the game. An addition, *2R* > *T* + *S* is required to ensure that cooperation exists. Table 1 shows the payoff matrix of the prisoner’s dilemma game.

### Calculation the payoff

Because an individual’s gain or loss depends on the actions of other players, the payoff to player *i* can be described using the payoff matrix of the prisoner’s dilemma game,


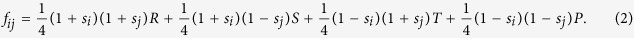


Here 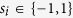 is the strategy of player *i*. If player *i* adopts a cooperation strategy, then 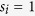, otherwise 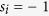. We focus on the weak prisoner’s dilemma^33^,^34^. For simplicity, we rescale the payoff matrix for the PDG to be *R* = 1, *S* = 0 and *P* = 0. We can also calculate the total payoff *f*_*i*_ of player *i* using Eq. (1),


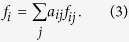


### Strategy updating rules

Because real-world individuals differ in their ability to acquire information, we divide players into two groups, those who are seeing and those who are blind. We assume that the initial proportion of seeing players is *p* and that they know both their own payoffs and the payoffs of each of their directly connected neighbors. Blind players only have knowledge of their own payoffs. A seeing player *i* decides whether to adopt the strategy of their neighbors. Player *i* with strategy *S*_*i*_ chooses neighbor *j* who has the maximum payoff and then imitates the strategy of *j*. We apply the Fermi rule that individual *i* adopts the strategy of selected neighbor *j* with a probability


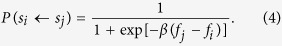


The quantity *β* ≥ 0, which in physics corresponds to the inverse temperature, here measures the strength of natural selection. When *β* = 0, we get 
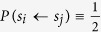
, which means that individuals are unaffected by payoff differences, and the probabilities of adopting the strategy of an individual with a high payoff or one with low payoff arethe same. Without losing generality, we set *β* = 10, which means that it is highly likely that the strategy of the better performing players will be adopted by their neighbors, and less likely (but not impossible) that the strategy of the less successful players will be adopted by their neighbors.

If players are blind, they will choose a strategy for the next round of the game based solely on their previous experience. If in the previous round *t*−1 player *i* strongly benefited using their chosen strategy, they will continue with that same strategy, but if in the previous round *t*−1 player *i* weakly benefited using their chosen strategy, they will adopt the opposite strategy. Typically player *i* randomly chooses a strategy that is either the same or the opposite of their previous strategy if there is no change in payoff. The adopted strategy of player *i* at game round *t* is


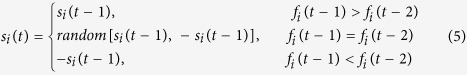


Here −*s*_*i*_ indicates a strategy opposite to *s*_*i*_, and *f*_*i*_(*t*−1) and *f*_*i*_(*t*−2) are the individual’s payoffs at game round *t* − 1 and *t* − 2, respectively.

### Nonparametric regression

We use nonparametric regression procedures to obtain a smooth set of points from each set of scattered data 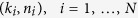. Nadaraya-Watson: we construct the kernel smoother function


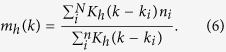


Here *K*_*h*_(*k* − *k*_*i*_) is a Gaussian kernel of the form,


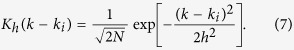


The optimal bandwidth *h* suggested by Bowman and Azzalini is


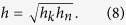


Where 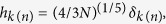 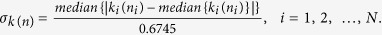


## Additional Information

**How to cite this article**: Li, D. *et al*. The co-evolution of networks and prisoner’s dilemma game by considering sensitivity and visibility. *Sci. Rep.* **7**, 45237; doi: 10.1038/srep45237 (2017).

**Publisher's note:** Springer Nature remains neutral with regard to jurisdictional claims in published maps and institutional affiliations.

## Figures and Tables

**Table 1:** The prisoner’s dilemma game payoff matrix.

	*C*	*D*
*C*	(*R, R*)	(*S, T*)
*D*	(*T, S*)	(*P, P*)
